# Adult nutrition and butterfly fitness: effects of diet quality on reproductive output, egg composition, and egg hatching success

**DOI:** 10.1186/1742-9994-5-10

**Published:** 2008-07-10

**Authors:** Thorin L Geister, Matthias W Lorenz, Klaus H Hoffmann, Klaus Fischer

**Affiliations:** 1 Department of Animal Ecology I, University of Bayreuth, D-95440, Bayreuth, Germany; 2 Zoological Institute & Museum, University of Greifswald, D-17489, Greifswald, Germany

## Abstract

**Background:**

In the Lepidoptera it was historically believed that adult butterflies rely primarily on larval-derived nutrients for reproduction and somatic maintenance. However, recent studies highlight the complex interactions between storage reserves and adult income, and that the latter may contribute significantly to reproduction. Effects of adult diet were commonly assessed by determining the number and/or size of the eggs produced, whilst its consequences for egg composition and offspring viability were largely neglected (as is generally true for insects). We here specifically focus on these latter issues by using the fruit-feeding tropical butterfly *Bicyclus anynana*, which is highly dependent on adult-derived carbohydrates for reproduction.

**Results:**

Adult diet of female *B. anynana *had pronounced effects on fecundity, egg composition and egg hatching success, with butterflies feeding on the complex nutrition of banana fruit performing best. Adding vitamins and minerals to a sucrose-based diet increased fecundity, but not offspring viability. All other groups (plain sucrose solution, sucrose solution enriched with lipids or yeast) had a substantially lower fecundity and egg hatching success compared to the banana group. Differences were particularly pronounced later in life, presumably indicating the depletion of essential nutrients in sucrose-fed females. Effects of adult diet on egg composition were not straightforward, indicating complex interactions among specific compounds. There was some evidence that total egg energy and water content were related to hatching success, while egg protein, lipid, glycogen and free carbohydrate content did not seem to limit successful development.

**Conclusion:**

The patterns shown here exemplify the complexity of reproductive resource allocation in *B. anynana*, and the need to consider egg composition and offspring viability when trying to estimate the effects of adult nutrition on fitness in this butterfly and other insects.

## Background

Availability of adequate nutrition is of crucial importance for successful reproduction. A multitude of studies documented pronounced effects of diet quality and quantity on female reproductive output in insects and thereby on fitness [1-3]. Fitness, however, is composed of various components, such that determining individual fitness is a challenging enterprise. Frequently, traits such as fecundity and/or egg size are used as proxies for individual fitness [1,4-8], while studies also taking offspring survival into account appear to be much rarer [9-13]. Results purely based on the former data might be misleading, as, for instance, egg size might be only vaguely related to fitness [10,11,14-18]. In order to gain a more integrated understanding of reproductive resource allocation, we need to shed more light on the interplay between reserves originating from storage versus income, between diet quality and egg composition, and the associated consequences for offspring viability.

Holometabolous insects are interesting models for studying reproductive resource allocation, because diets and energetic needs change dramatically between life stages, warranting integrated strategies for timed nutrient accumulation, storage and release (e.g. [1,19-22]). This seems particularly important for female insects, since nutrient investment into eggs constitutes a major expenditure of energy [1,19,23]. The Lepidoptera, feeding as larvae on protein-rich plant foliage, were historically believed to primarily rely on larval-derived nutrients for reproduction and somatic maintenance [2,24-27]. However, recent studies highlight the complex interactions between storage reserves and adult income, and that the adult diet may contribute significantly to reproductive output (e.g. [2,25,28-31]). Even other substrates ranging from pollen to mud, dung or carrion may comprise important sources of scarce nutrients (e.g. [32-35]). While substantial progress was made in some of these areas in recent years (especially in relation to use of income versus storage [24-26,28,30,36]), others remained poorly understood. This is particularly true for the effects of adult nutrition on egg composition and in turn on offspring fitness [10,16,37-39].

In this study we draw on these under-explored areas of insect reproduction by using the tropical, fruit-feeding butterfly *Bicyclus anynana *as model organism. In this species adult-derived carbohydrates are essential for egg production, without which no eggs will be produced ("income breeder" [30,40]). Consequently, even the first eggs laid contain considerable amounts of adult-derived nutrients, followed by a quick shift towards even higher ratios [30]. Note in this context that fruit is not necessarily a richer source of nutrients than nectar, at least in terms of carbohydrate and nitrogen content [41-44]. However, fruit may contain considerable amounts of lipids and a variety of micronutrients that may also benefit reproduction [45]. Recent studies using this species showed that females have a significantly higher reproductive output and longer life-spans when fed with banana as compared to sugar-based diets [40,46]. As various compounds such as lipids or amino acids had no positive effects on the above traits, it was concluded that the beneficial effects of fruit are caused by resource congruence (i.e. the use of nutrient types in a specific ration [47]) rather than any specific compound [40,46]. In addition to reproductive output, here we investigate the effects of adult diet on egg composition and egg hatching success. Specifically we examine whether adult diet has a direct impact on egg composition (such that e.g. a lipid-rich diet causes an increased lipid content of eggs), and whether any potential variation in egg content is related to offspring viability.

## Results

### Fecundity, egg size and hatching success

**Figure 1:**
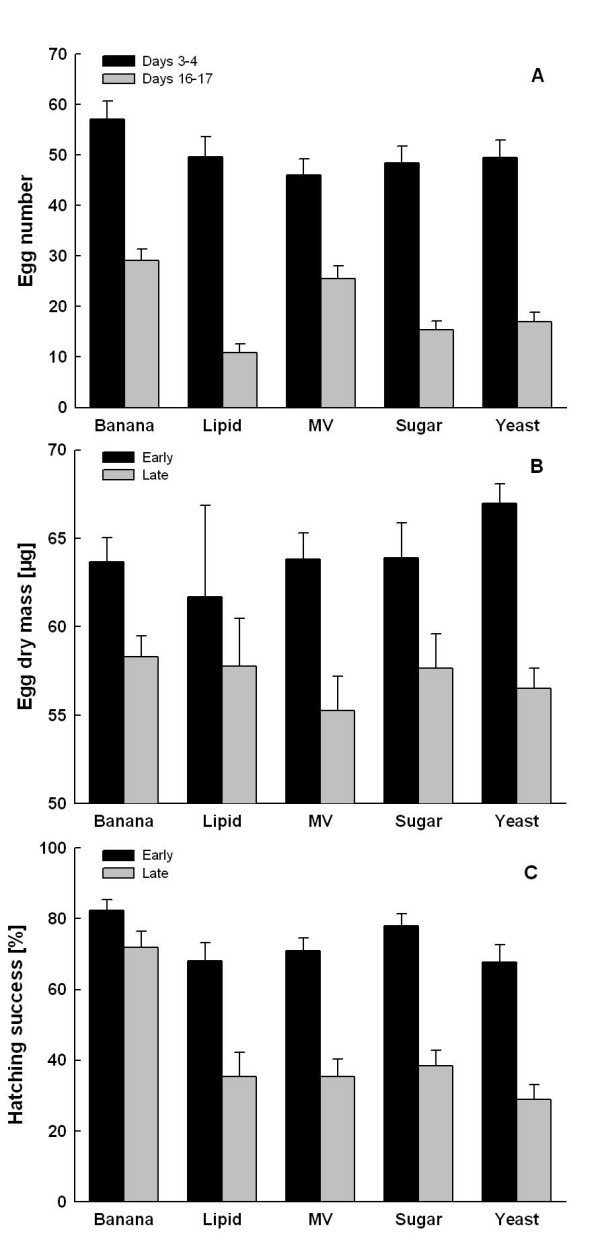
**Egg number, dry mass and egg hatching success**. Mean egg number (A), egg dry mass (B), and egg hatching success (C) for female *Bicyclus anynana *in relation to adult feeding and female age. Early: Eggs from days 3–4 of adult life; late: eggs from days 16–17. Banana: moist banana; Lipid: 20 Vol% sucrose plus 1 Vol% olive oil; MV: 20 Vol% sucrose plus minerals and vitamins; Sugar: 20 Vol% sucrose; Yeast: 20 Vol% sucrose plus baker's yeast.

Adult diet significantly affected fecundity of *B. anynana *females (*F*_4,250 _= 6.1, *P *< 0.001). The banana group achieved the highest egg numbers (86.2 ± 4.5) followed by the MV group (71.6 ± 4.1), while the remaining treatment groups laid significantly fewer eggs and were statistically indistinguishable (63.8 ± 2.3; Tukey HSD after ANOVA; Fig. 1). While early fecundity was fairly similar across treatment groups (except from slightly higher values for the banana group), variation in late fecundity was higher with the banana and MV groups showing substantially higher values than all other groups (significant treatment × time interaction; *F*_4,250 _= 2.5, *P = *0.041). Generally, females produced much more eggs early compared to late in life (*F*_1,250 _= 256.3, *P *< 0.001). Egg dry mass did not differ significantly across treatment groups (*F*_4,129 _= 0.260, *P *= 0.903), but was overall significantly lower (reduction by ca. 11%) later than earlier in life (*F*_1,129 _= 23.8, *P *< 0.001; treatment × time interaction: *F*_4,129 _= 0.66, *P *= 0.624; Fig. 1).

**Figure 2:**
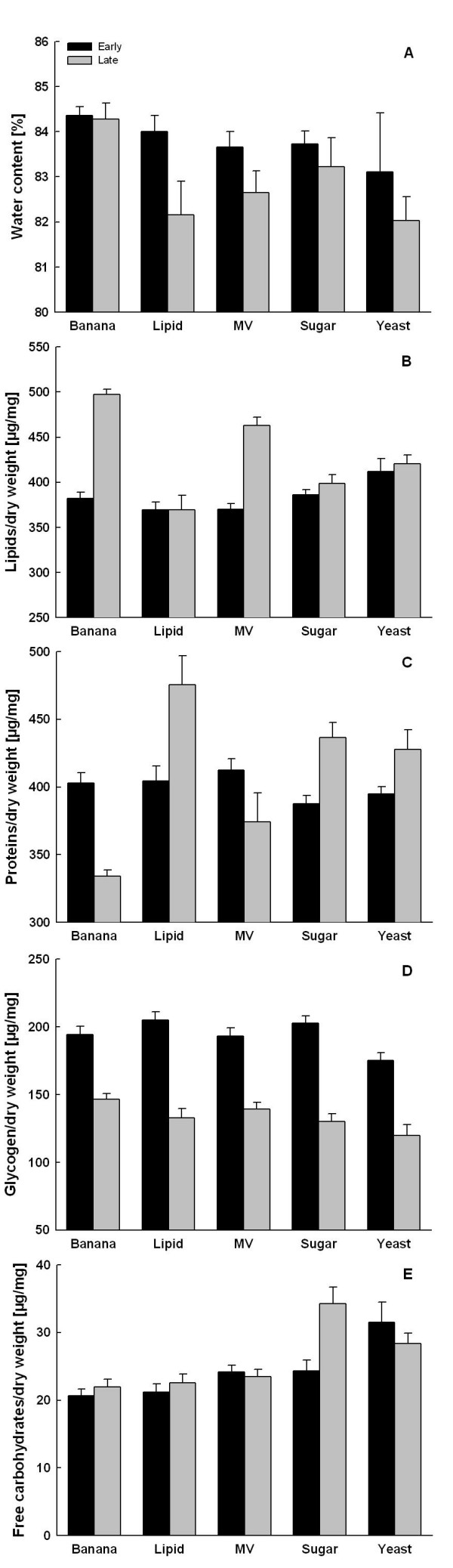
**Relative egg composition**. Relative egg water (A), lipid (B), protein (C), glycogen (D) and free carbohydrate content (means ± 1 SE) for female *Bicyclus anynana *in relation to adult feeding and female age. Early: Eggs from days 3–4 of adult life; late: eggs from days 16–17. For explanations of dietary treatments see Fig. 1.

Egg hatching success was overall significantly higher in the banana compared to all other groups, with the latter four groups being statistically indistinguishable (Tukey HSD; *F*_4,213 _= 10.8, *P *< 0.001; Fig. 2). The overall better performance of the banana group resulted mainly from a constantly high hatching success (early life: 82.4 ± 3.6%, late life: 71.9 ± 4.6%), whilst in the other treatment groups hatching success dropped markedly from ~70% (early) to ~30% (late; time: *F*_1,213 _= 177.3, *P *< 0.001; treatment × time: *F*_4,213 _= 6.2, *P *< 0.001; Fig. 2).

### Egg composition

**Figure 3:**
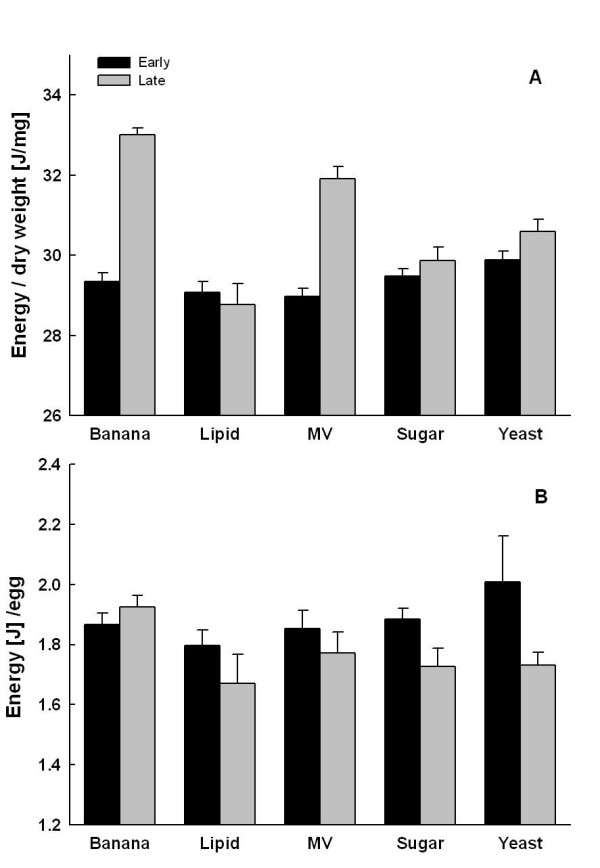
**Egg energy content**. Egg energy content per 1 mg dry mass (A) and per egg (B) (means ± 1 SE) for female *Bicyclus anynana *in relation to adult feeding and female age. Early: Eggs from days 3–4 of adult life; late: eggs from days 16–17. For explanations of dietary treatments see Fig. 1.

Across treatments and sampling periods (i.e. for eggs produced early or late in life), eggs consisted predominantly of water (84.1 ± 0.24%), followed by lipids (6.8 ± 0.11%), proteins (6.5 ± 0.19%), glycogen (2.1 ± 0.05%) and free carbohydrates (0.4 ± 0.02%). Egg water content was significantly higher in the banana than in the yeast group, while all other pairwise comparisons were not significant (Tukey HSD after ANOVA; *F*_4,129 _= 2.5, *P *= 0.049; Fig. 3). Overall, water content was significantly lower in later than in earlier eggs (*F*_1,129 _= 5.1, *P = *0.026; treatment × time interaction: *F*_1,129 _= 0.6, *P = *0.677). Adult diet significantly influenced egg lipid (*F*_4,131 _= 13.4, *P *< 0.001; Fig. 3), protein (*F*_1,129 _= 10.7, *P *< 0.001; Fig. 4), glycogen (*F*_4,131 _= 4.1, *P *< 0.001; Fig. 4) and free carbohydrate content (*F*_4,130 _= 10.1, *P *< 0.001; Fig. 5).

Lipid content was higher in the banana and MV groups compared to all other treatments. Further, lipid content increased with female age (*F*_4,130 _= 62.0, *P *< 0.001), particularly in the banana and MV groups (treatment × time interaction: *F*_4,130 _= 17.7, *P *< 0.001). Protein content, in contrast, was significantly lower in the banana and MV than in the other groups. While protein content decreased with female age in those two treatment groups, it increased in the remaining three groups (treatment × time interaction: *F*_4,130 _= 19.0, *P *< 0.001). Accordingly, no overall time effect for protein content was observed (*F*_2,130 _= 2.5, *P *= 0.129).

Gycogen content was significantly reduced in the yeast group and was further significantly reduced in late compared to early eggs (*F*_4,131 _= 365.7, *P *< 0.001; treatment × time interaction: *F*_4,131 _= 2.2, *P *= 0.071). Free carbohydrate content was significantly higher in the sugar and yeast treatments. While free carbohydrate content was generally rather constant over time (*F*_4,131 _= 3.1, *P = *0.082), it strongly increased with female age in the sugar group, but slightly decreased in the yeast group (treatment × time interaction: *F*_4,131 _= 5.3, *P *< 0.001).

Across both sampling periods and all treatments egg lipid content was rather strongly negatively correlated to protein content (i.e. the higher the lipid, the lower the protein content and vice versa; Table 2). Further, glycogen tended to be negatively related to protein content (significant in 8 out of 10 cases). Correlations between lipid and glycogen content as well as free carbohydrates to the other three content groups revealed no conclusive pattern.

**Table 2:** Inter-correlations between egg compounds.

		**Early**			**Late**		
	*N*	Lipid Protein	Glycogen Protein	Lipid Glycogen	Lipid Protein	Glycogen Protein	Lipid Glycogen

Banana	29	*** - 0.69	*** - 0.60	- 0.15	*** - 0.62	- 0.35	*** - 0.47
Lipid	18	*** - 0.82	* - 0.55	-0.01	*** - 0.96	*** - 0.76	* + 0.56
MV	30	*** - 0.60	*** - 0.64	- 0.20	*** - 0.87	* - 0.57	-0.12
Sugar	30	*** - 0.64	** - 0.56	- 0.23	*** - 0.84	*** - 0.63	+ 0.14
Yeast	29	*** - 0.56	- 0.33	*** - 0.53	*** - 0.80	*** - 0.76	+ 0.23

		Carbohydrates Protein	Carbohydrates Lipid	Carbohydrates Glycogen	Carbohydrates Protein	Carbohydrates Lipid	Carbohydrates Glycogen

Banana	29	+ 0.04	- 0.32	+ 0.13	+ 0.01	* - 0.37	+ 0.21
Lipid	18	- 0.35	+ 0.27	+ 0.04	* - 0.47	+ 0.43	+ 0.26
MV	30	** - 0.53	+ 0.19	+ 0.35	+ 0.08	- 0.23	+ 0.05
Sugar	30	+ 0.21	- 0.35	- 0.18	+ 0.31	**- 0.57	- 0.04
Yeast	29	* - 0.45	- 0.04	+ 0.12	*** - 0.60	+ 0.26	*** + 0.61

Inter-correlations between *Bicyclus anynana *egg compounds per dry weight in relation to adult feeding treatment and time. Early: Eggs from days 3–4 of adult life; late: eggs from days 16–17. Given are the correlation coefficients. * *P *< 0.05, ** *P *< 0.01, *** *P *< 0.001.

### Energy investment

Energy investment per 1 mg egg dry mass was significantly higher in banana and MV females compared to other treatments (Tukey HSD after ANOVA; *F*_4,128 _= 15.4, *P *< 0.001), and was higher in eggs laid later in life than in those laid earlier (*F*_1,128 _= 83.4, *P *< 0.001; Fig. 6). The latter effect was particularly pronounced in the banana and MV treatments (treatment × time interaction; *F*_4,128 _= 22.7, *P *< 0.001). Energy investment per egg, in contrast, did not differ significantly between treatments (*F*_4,128 _= 1.2, *P *= 0.337), but was significantly lower (by about 6%) in eggs oviposited later in life (*F*_1,128 _= 6.9, *P *< 0.001; treatment × time interaction: *F*_1,128 _= 1.7, *P *= 0.156; Fig. 6). Across treatment groups, egg dry mass was significantly positively related to egg energy content (early: *r *= 0.98, *P *< 0.001, n = 135; late: *r *= 0.93, *P *< 0.001, n = 136).

## Discussion

### Fecundity, egg size and hatching success

Female *B. anynana *butterflies feeding on the complex nutritional diet of banana showed highest fecundity levels, followed by the group fed a sugar solution supplemented with minerals and vitamins (MV). The higher average egg numbers in these groups mainly reflect a much less pronounced reduction in daily fecundity with female age compared to the other groups. The general decline in egg numbers with female age in turn probably reflects the depletion of essential (larval-derived) resources [14,16]. Thus, these results show, in line with previous findings, that banana is the superior food in terms of egg numbers, with only the solution containing vitamins and minerals resulting in an increased fecundity (above the level of a plain sugar solution [30,40,46]), whereas adding nutrients to banana diet, does not necessarily rise fecundity [48].

Since insect eggs are primarily composed of protein and lipid [38,49-52], we anticipate a high demand for these compounds by ovipositing females, and yeast is known to be an excellent source of protein to insect frugivores [53]. Furthermore lipids are expected to be invaluable since insects are known to be able to produce the triacylglycerol needed for egg production from the free fatty acids provided by our lipid treatment, and also to transport those into developing oocytes [51,54,55]. However, neither the yeast nor the lipid treatment affected reproductive output of *B. anynana*, so we conclude that female reproduction is usually not constrained by adult-derived lipid or protein [24,25,40,46].

On the other hand, the evidence suggests that females may eventually run out of vitamins or minerals carried over from the larval stage during oviposition. Interestingly, adding either minerals or vitamins to the adult diet had no effect on *B. anynana *fecundity, but only the combination of both [40]. Positive effects of additionally providing minerals or vitamins, though not necessarily in combination with each other, on fecundity have been reported also for some other insects [49,56]. Generally, however, the role of those compounds in insect reproduction is only poorly understood, while their importance for insect energy metabolism, growth, development or detoxication is rather well established (e.g. [57-59]).

In contrast to fecundity, egg size was not affected by the manipulation of diet quality, although former studies reported slightly larger eggs laid by females fed banana compared to other diet groups [40,60]. Anyway, when manipulating diet quality or other factors, variation in egg size was generally found to be much less pronounced as compared to fecundity [40,46,61,62]. The decrease in egg size with female age is a common feature repeatedly documented for a variety of butterfly species (e.g. [1,14,63,64], but [65]). In addition to producing most eggs, banana-fed females also had the highest egg hatching success, again documenting the high quality of this adult diet. While early in life hatching success was very similar across feeding treatments, it dropped dramatically later in life except for the banana group. These findings suggest the depletion of one or more crucial substances in the females fed sucrose-based diets, and that the respective compounds can obviously be drawn from the fruit diet. We do not know the compounds in question, but can largely rule out proteins, lipids, and the minerals and vitamins tested. Most likely this pattern is caused by resource congruence rather than by any specific compound (note that hatching success dropped substantially even in the MV treatment), as has been implicated before with regard to reproductive output [40,46]. While a substantial number of studies examined the effects of larval and adult nutrition on reproductive output, very little is known on the effects of adult nutrition on offspring fitness [10,39]. In the rove beetle *Tachyporus hypnorum*, for instance, maternal diet affected larval development and offspring survival [10], while in the dung fly *Scathophaga stercoraria *low adult food quality had no effect on egg hatching success [39]. In the next section we discuss the associations between offspring fitness and egg composition which might causally underlie the variation in hatching success found in our study.

### Egg composition

Generally, egg size and composition are not necessarily tightly correlated, and variation in egg composition can be ecologically and evolutionarily more important than variation in egg size [5,15,16,66]. In line with other data on insects [51,67], *B. anynana *eggs consist mainly of water, proteins and lipids. An egg water content of about 84% is typical for satyrine butterflies [68]. Highest values were found in the banana, lowest in the yeast group. The latter may reflect dehydration associated with the production of ethanol by yeast [69]. Egg water content was generally lower in late than in early eggs, except for the banana group (but note that the interaction is not significant [38]). Although water content variation seems to be small based on percentages, such differences confer to a rather large part of fresh egg weight, especially as egg dry mass is only about 16%. As the banana group is the only one maintaining a high egg hatching success later in the oviposition period, egg water content might have a significant impact on egg hatching success (compare Figs 2 and 2A; see below).

Compared to all other treatments, the eggs produced by banana and MV females showed higher amounts of lipids, but lower amounts of proteins. In both groups this pattern is primarily caused by variation in the later eggs. While relative lipid content remained largely similar over time in the sugar, yeast and lipid group, it increased dramatically in late compared to early eggs in the banana and MV group. Similar results were obtained from selection lines, which were selected for differences in egg size [38]. Relative protein content decreased with female age in the banana and MV group, but it increased in the remaining groups. The similarity of patterns across both groups is striking and may suggest that the increased fecundity of the banana and MV groups caused a gradual depletion of nitrogenous compounds, compensated by an increased investment of lipids. Likewise, a reduction in protein investment with female age was found in *B. anynana *females having been exposed to larval food stress [38]. It is generally assumed that the vast majority of nitrogenous compounds used for reproduction is accumulated and stored during the larval phase (e.g. [22,24,26]), though adult diet may in some cases compensate larval deficiencies [25].

The general importance of lipids and proteins for embryonic and larval development in insects is well established [11,67,70]. Lipids are considered to cover the energetic demands of the developing embryo, while proteins are mainly structural components, but may additionally serve as energetic resource [70]. Furthermore, yolk protein has been previously shown to be a good predictor for neonate fitness [11]. Nevertheless, neither compound had a detectable influence on egg hatching success in *B. anynana*. Despite a strong reduction of relative protein content in late eggs, banana-fed females were able to maintain egg hatching success on a high level, while the increased investment of protein in other groups could not prevent a reduction in egg viability. Likewise hatching success of later eggs in the MV group was as low as for the other groups (except banana), despite a clearly increased relative lipid content.

Glycogen content was similar across treatments except for the yeast group, but was considerably lower in late compared to early eggs (see also [38]), probably reflecting the onset of stored glucose usage for reproduction [71]. Free carbohydrate levels in the hemolymph are known to increase with carbohydrate-rich diet [72,73]. Here, free carbohydrate content in the eggs was higher in the sugar and yeast groups, although all treatments should have provided sufficient amounts of free carbohydrates. In all other treatments free carbohydrates were similar in early and late eggs, at it has been reported before [38]. Neither glycogen nor free carbohydrates showed any obvious association with fecundity or hatching success.

The negative association between lipid and protein across adult diet groups described above is also found within groups: throughout, strong negative correlations between both compounds were found. Furthermore, glycogen tended to be negatively related to protein content. These findings may suggest a trade-off between the investment into structural elements and storage reserves.

### Energy investment

Estimates of reproductive investment often exclusively rely on egg numbers and/or egg size [4,5,15]. This approach is problematic as egg size can be uncorrelated with energy content [18], as individual egg compounds may vary independently of egg size [17], and as energetic investment may decrease with female age [16,74]. Nevertheless studies on insects taking the actual energetic investment into account (by means of biochemical analyses) are scarce [11,13,16,37,67]. In *B. anynana *egg size correlated strongly with energy content, indicating that eggs size can be a fair proxy of energetic investment into reproduction. Nevertheless individual compounds showed substantial variation, suggesting that allocation strategies may differ in spite of similar egg size and energy content.

Investment per 1 mg dry mass followed, as expected, patterns of variation in lipid content. Consequently eggs from the banana and MV groups showed an overall higher relative energy investment, especially so in late eggs. It is noteworthy that relative investment was not reduced in later eggs in any group. In contrast, absolute investment per egg was similar across treatment groups, but was ca. 6% lower in later as compared to earlier eggs (except for the banana group). This can be largely attributed to variation in egg dry mass, declining by ca. 11%. The association between a high energetic investment even in late eggs and high hatching success found in the banana group suggests that egg energy content might be crucial for egg viability (see below).

While the general decline in egg provisioning with female age (egg size, absolute energy content) is in agreement with other studies (e.g. [1,14-16,74]), relative investment (per mg dry mass) as well as absolute investment in animals fed with a high quality food remained unaffected (see also [38]). The latter was achieved, despite a substantial decline in egg size, by increasing relative energy investment (i.e. provisioning with lipids). Thus, caution is needed when trying to draw general conclusions, and studies on reproductive resource allocation should take into account variation in egg quality, hatching rate and larval survival [15].

## Conclusion

Individual fitness is a complex trait that is difficult to measure. A commonly used method for estimating the fitness effects of dietary treatments (and other factors) is determining the number (and sometimes size) of eggs produced (see [4,5]). Studies directly measuring offspring viability, in contrast, are much less frequent [11-13,15],. and very little is known to date on the interplay between diet quality, age, egg content, and offspring viability [15,37,38]. Our study provides some insights regarding the latter. Clearly, the complex nutritional composition of banana fruit was superior compared to alternative diets, not only increasing reproductive output compared to sugar-based diets [40,46], but also positively affecting egg hatching success. As has been argued before [40,60] we believe the increased reproductive output to be a consequence of resource congruence, as sugar enriched with minerals and vitamins increased fecundity (though not reaching the levels of the banana group), while either adding protein or lipids had no detectable effect.

Adult diet had striking effects on egg composition, namely on water, protein, lipid and carbohydrate content. The effects, however, were not straightforward (e.g. lipid content was not increased in lipid group, and protein content was not increased in the yeast group), indicating complex interactions among specific compounds (see also [48]). Protein content declined with female age, but only in the groups exhibiting highest fecundity (banana, MV), suggesting that *B. anynana *reproduction strongly depends on nitrogenous resources accumulated during the larval phase (see also [22,24,26]). This development was counter-balanced by an increased investment of lipids into later eggs, presumably synthesised from the adult diet [30,61]. Despite the overall pronounced variation in egg composition, no single compound showed any clear association with egg hatching success, and neither did egg size (note the high hatching success for the relatively small eggs produced late by banana-fed females).

Only two traits showed at least some evidence for an association with offspring viability: absolute energy content and egg water content. Both compounds were present in largely similar quantities in early and late eggs from the banana group (being associated with a high hatching success throughout), while they clearly decreased with female age in all other groups, followed by a decrease in hatching success. We tentatively conclude that egg protein content is not limiting in *B. anynana*, possibly based on a minimum threshold beyond which no successful development is possible. This threshold may not have been touched here. Note that protein content in *B. anynana *was remarkably stable across eggs artificially selected for large and small size [38]. Rather, the amount of energy available for embryonic development and egg water content may determine hatching success. There is some evidence already that a high water content, presumably reducing desiccation risk, may be important for successful egg development in *B. anynana *[75,76]. Consequently, water should not be exclusively considered a cheap filler, also since probably some energy is needed to incorporate water into eggs. As *B. anynana *experiences a wet and a dry season in its natural environment, water content might be a critical factor especially during the dry season, in which development times are rather long, and temperature and humidity is rather low [77,78]. During this season the rather small absolute differences in water content may well be of ecological relevance, especially as differences were more pronounced later during the oviposition period. While dry season butterflies show delayed oviposition, selection appears to favour rapid reproduction during the wet season [75,79].

The importance of adult diet for different components of *B. anynana *fitness exemplifies the complexity of reproductive resource allocation in insects, which were formerly assumed to rely primarily on larval stores [2,27]. Clearly, more such efforts are needed before general conclusions on the effects of adult diet on egg composition and the role of specific compounds for egg hatching success can be drawn.

## Methods

### Study organism and experimental population

For this study the tropical butterfly *Bicyclus anynana *Butler, 1879 (Lepidoptera, Nymphalidae, Satyrinae) was used. *B. anynana *is a fruit-feeding butterfly with a distribution ranging from Southern Africa to Ethiopia, which feeds on a variety of fallen and decaying fruit [77,80]. A laboratory stock population was established at Bayreuth University, Germany, in 2003 from several hundred individuals derived from a well-established stock population at Leiden University, The Netherlands. The Leiden population was founded in 1988 from over 80 gravid females caught at a single locality in Malawi. Several hundred adults are reared in each generation, maintaining high levels of heterozygosity at neutral loci [81]. Animals from the Bayreuth stock were used for this study.

### Experimental design

To investigate the effects of adult nutrition on reproductive output, egg composition and egg hatching success all *B. anynana *individuals were reared within the same environmental cabinet at a constant temperature of 27°C, high relative humidity (70%) and a photoperiod of L12:D12. Larvae originating from several hundred fecund butterfly females were reared on young maize plants in population cages (50 × 50 × 80 cm), with plants being regularly replaced. The resulting pupae were collected from the plants and transferred to cylindrical hanging cages (30 × 38 cm). Following adult eclosion, female butterflies were randomly divided among five nutritional treatments: 1) moist banana (hereafter 'banana', for the general composition of banana see [45]), 2) sucrose solution (20 Vol%; 'sugar'), 3) sucrose solution (20 Vol%) enriched with lipids (1 Vol% olive oil [Roth, Karlsruhe, Germany], containing ~14% 16:0 palmitic acid, ~3% 16:1 palmitoleic acid, ~3% 18:0 stearic acid, ~70% 18:1 oleic acid, ~12% 18:2 linoleic acid and 18:3, 20:0, 20:1, 22:0, 22:1 acids < 0.1 Vol%; 'lipid'), 4) sucrose solution (20 Vol%) enriched with a combination of minerals and vitamins readily found in banana (c.f. [40]; Table 1; 'MV) and 5) a sucrose solution (20 Vol%) enriched with baker's yeast (*Saccharomyces cerevisiae*; ~0.5 Vol%; 'yeast'). The sucrose solution enriched with lipids was prepared by using ultrasound with the resulting suspension being clearly stable for one day after which diets were generally renewed. For the yeast treatment, commercial baker's yeast was dissolved in the appropriate sugar solution. As all butterflies were facing no-choice situations and *B. anynana *butterflies are used to feed on a variety of different food sources, it is highly unlikely that effects are based on different rates of intake. Furthermore, preference tests revealed that *B. anynana *responds exclusively to sugars (and alcohols) but not to any other of the admixtures used here (in preparation), and different sucrose solutions did not influence the longevity of *B. anynana *[40].

**Table 1:** Provided minerals and vitamins.

**Minerals**			
Potassiumchloride	3900 mg	Magnesiumchloride	360 mg

**Vitamins**			

Retinolequivalent (Vitamin A)	4.6 mg	Pantothenic acid (Vitamin B5)	4.6 mg
Thiamine (Vitamin B1)	0.9 mg	Pyridoxine (Vitamin B6)	7.4 mg
Riboflavin (Vitamin B2)	1.1 mg	Ascorbic acid (Vitamin C)	240 mg
Niacinequivalent (Vitamin B3)	19.0 mg		

Amounts of minerals and vitamins per 1 L 20 Vol% sucrose solution for the feeding treatment sucrose solution enriched with minerals and vitamins (MV).

On the day after eclosion, all females were set up for mating with random males for two days. All male butterflies were fed moist banana, as their contribution to reproduction (e.g. transfer of sodium via spermatophores) is considered to be negligible [82-84]. Following the mating period, females were marked individually and placed into translucent plastic pots (1 L, covered with gauze), containing a fresh cutting of maize for egg-laying. Females were provided with their described nutritional treatments throughout their lives.

As a proxy for early and late fecundity the eggs laid on days 3 and 4 and 16 and 17 of adult life were collected and counted. The eggs laid on the first day of oviposition (i.e. day 3) were discarded, as they contain relatively high amounts of larval resources (see [30]), while the focus of the present study is on adult-derived resources. The first period represents the beginning of the oviposition period in *B. anynana *with maximum daily egg numbers. Under experimental conditions at 27°C, maximum life-spans are around ~30 days, with nearly half of the butterflies being dead around days 16/17 [40,85]. Per female, 20–40 eggs from each collection period were randomly divided into two groups: one half was used for recording egg hatching success, whereas the other half was stored at -20°C for later analysis of egg composition. For the early collection period 1.9 ± 0.08 additional days were necessary to collect enough eggs for further experiments, while for the later +3.9 ± 0.12 days were needed. The two time points were chosen as differences in adult diet may take some time to take effect, i.e. any potential differences might be hidden at the beginning of the oviposition period due to overriding effects of larval storage reserves.

### Egg hatching success

Eggs were separated by female, transferred to Petri dishes and reared at the same conditions described above. Hatchlings were counted and removed daily until no more larvae had hatched for at least two days. Finally, remaining eggs were counted and the proportion of eggs hatched was calculated.

### Egg composition

Only females having laid ≥ 20 eggs during both collecting periods were used for egg content analyses. If more eggs were available, a maximum of 20 randomly chosen eggs were used. To rule out confounding effects of (potentially occurring) egg shell contamination with dietary fluids, eggs were washed prior to analyses as follows: egg samples were put into 250 μl H_2_O, washed for 10 sec. in an ultrasonic bath, transferred to 500 μl CHCl_3_:MeOH (1:1), and washed again for 10 sec. in an ultrasonic bath. Then, the solvent was removed using a borosilicate filter membrane on a vacuum pump, and the samples were rinsed with 1 ml H_2_O. The latter procedure was repeated once. Before washing, egg fresh mass was determined, and after the washing procedure eggs were dried for 24 h at 70°C and weighed again. Egg water content was estimated as mass difference between egg fresh and dry mass. The extraction and separation of egg lipid, protein, glycogen, and free carbohydrate from the same samples was carried out as described by Lorenz [52]. Colorimetric determination of total lipid, glycogen, and free carbohydrate was performed using modified sulphophosphovanillin and anthrone methods. Protein was measured with an EL 808 Ultra Microplate Reader (Bio-Tek Instruments, Inc., Winooski, Vermont, USA) using the RotiQuant Universal assay (Roth, Karlsruhe, Germany) and bovine serum albumin as a standard [38,52]. Throughout, data were corrected using measured recovery rates [free carbohydrate: 96.5 ± 2.4%, lipid: 91.1 ± 2.1%, protein: 72.7 ± 2.6%, glycogen: 83.4 ± 1.9%, 52]. To correct for different recovery rates between treatments (due to variation across days of analysis), data were standardized to 100% dry mass. From the resulting values for egg components, energy investment per mg egg dry mass was calculated using average caloric values of 17.2 kJ g^-1 ^for free carbohydrates, proteins and glycogen and 39.0 kJ g^-1 ^for lipids [86,87].

### Statistical analysis

Data were analyzed using repeated measurement ANOVAs with the nutritional treatments as categorical factor and both collecting periods as repeated measures. In case of a global significance, differences between treatment groups were localized using Tukey's HSD. Pearson correlations were used to analyze the relationships between different egg components. All statistical tests were performed using STATISTICA 6.1. Throughout the text, means are given ± 1 SE.

## Competing interests

The authors declare that they have no competing interests.

## Authors' contributions

TLG, KF, MWL and KHH designed the research, TLG performed the experiments, analysed the data and wrote the manuscript together with KF. All authors read and approved the final manuscript.
